# Simultaneous Strength-Ductility Enhancement of a Nano-Lamellar AlCoCrFeNi_2.1_ Eutectic High Entropy Alloy by Cryo-Rolling and Annealing

**DOI:** 10.1038/s41598-018-21385-y

**Published:** 2018-02-19

**Authors:** T. Bhattacharjee, I. S. Wani, S. Sheikh, I. T. Clark, T. Okawa, S. Guo, P. P. Bhattacharjee, N. Tsuji

**Affiliations:** 1 Department of Materials Science and Engineering, Kyoto University, Kyoto, Japan; 2 Department of Materials Science and Metallurgical Engineering, IIT Hyderabad, Hyderabad, India; 3 Industrial and Materials Science, Chalmers University of Technology, SE-41296 Gothenburg, Sweden; 4 Scienta Omicron, Inc, 6-16-4 Minami-Oi, Shinagawa-ku, Tokyo, 140-0013 Japan; 5 Elements Strategy Initiative for Structural Materials (ESISM), Kyoto University, Kyoto, Japan

## Abstract

Nano-lamellar (L1_2_ + B2) AlCoCrFeNi_2.1_ eutectic high entropy alloy (EHEA) was processed by cryo-rolling and annealing. The EHEA developed a novel hierarchical microstructure featured by fine lamellar regions consisting of FCC lamellae filled with ultrafine FCC grains (average size ~200–250 nm) and B2 lamellae, and coarse non-lamellar regions consisting of ultrafine FCC (average size ~200–250 nm), few coarse recrystallized FCC grains and rather coarse unrecrystallized B2 phase (~2.5 µm). This complex and hierarchical microstructure originated from differences in strain-partitioning amongst the constituent phases, affecting the driving force for recrystallization. The hierarchical microstructure of the cryo-rolled and annealed material resulted in simultaneous enhancement in strength (Yield Strength/YS: 1437 ± 26 MPa, Ultimate Tensile Strength/UTS: 1562 ± 33 MPa) and ductility (elongation to failure/e_f_ ~ 14 ± 1%) as compared to the as-cast as well as cold-rolled and annealed materials. The present study for the first time demonstrated that cryo-deformation and annealing could be a novel microstructural design strategy for overcoming strength-ductility trade off in multiphase high entropy alloys.

## Introduction

Increasing strength of materials (σ) leads to a reduction in cross-sectional area (A) for bearing a certain load (P) according to the definition of stress: $\sigma =P/A$. This provides the necessary impetus for utilizing ultrahigh strength materials for making engineering components with thinner cross-sections; leading to significant weight savings with immense environmental benefits. Unfortunately, however, the increase in strength is usually accompanied by the reduction in ductility. This so called “strength-ductility trade-off” remains a bottleneck^1^. The strategies for simultaneously increasing strength and ductility in metallic materials have mostly adopted novel alloy design approaches, including martensitic transformation from metastable austenite (e.g. TRIP steels) or by way of promoting extensive deformation twins (e.g. TWIP steels)^2,3^. More recently, microstructure design approach including gradient structures^4–10^, heterogeneous lamella structures^11^, bimodal structures^12–15^, harmonic structures^16–18^, laminate structure^19,20^, nano-domained^21^, nano-twinned grains^22^ and compositional nanoscale hierarchy^23,24^. These microstructure design strategies are based on hierarchical architecture featured by differences in hardness and strength between different domains, while the size and geometry of the domains might be very different. Thus, significant heterogeneities (microstructural, crystal structure or compositional) are present in such materials which appear to be the key factors for overcoming the strength-ductility trade-off.

Recently, high entropy alloys (HEAs) have emerged as a novel class of multicomponent alloys containing five or more elements in equi-atomic or nearly equi-atomic compositions^25^. Despite having a large number of components, HEAs can show simple phases such as FCC, BCC and FCC + BCC^25,26^, presumably due to their high configurational entropy, which decreases the free energy sufficiently to stabilize simple solid solution phases. HEAs have triggered a remarkable research interest in recent years^26–30^ due to their unique and intriguing mechanical properties^27,28,31–33^.

In order to further enhance the properties of HEAs, dual and multi-phase HEAs having mixture of soft and hard phases are suggested. Non-equiatomic AlCoCrFeNi_2.1_ eutectic HEA is a noteworthy example of such multiphase HEAs^34^. Wani *et al*.^35,36^ have recently demonstrated that thermo-mechanical processing can effectively tailor the microstructure and properties of the EHEAs. In the present work, an interesting effect of cryo-rolling and annealing on the microstructure and mechanical properties of AlCoCrFeNi_2.1_ EHEA is reported. Although cryo-rolling has been investigated in single phase HEAs^37,38^, we have found a remarkable effect of cryo-rolling on tensile properties of the EHEA for the first time.

## Results

**Figure 1:**
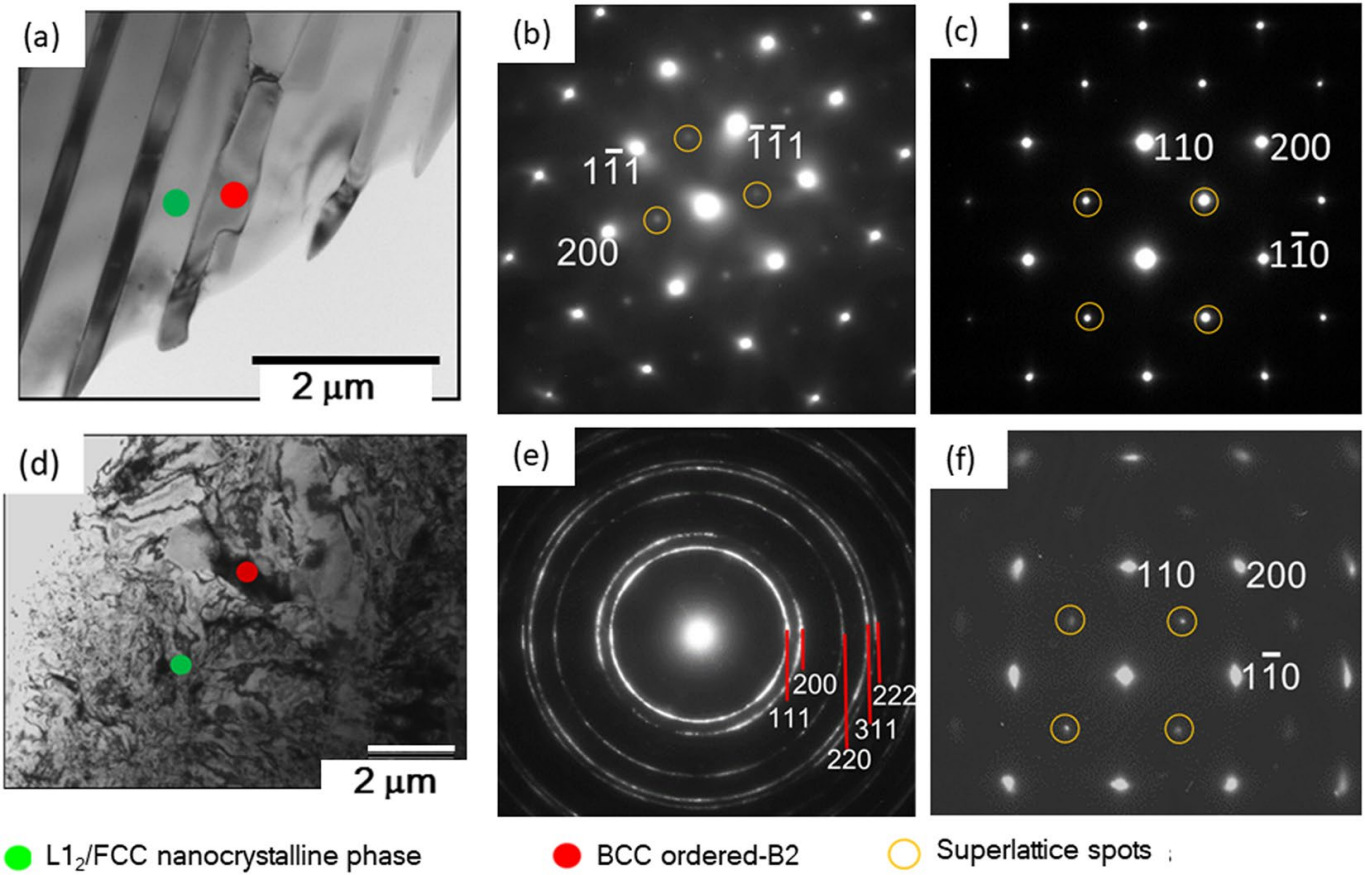
(**a**) TEM micrograph of the starting EHEA. The SADPs in (**b**) and (**c**) correspond to the L1_2_ (marked by green circle in (**a**)) and B2 (indicated by red circle in (**a**)), respectively. The zone axes (Z.A.) of the L1_2_ and B2 phases are [011] and [001], respectively. (**d**) Shows the microstructure in the 90% cryo-rolled condition (viewed along ND). The SADPs in (**e**) and (**f**) correspond to the nanocrystalline disordered FCC (indicated by the green circle in (**d**)) L1_2_ (marked by green circle in (**d**)) and B2 (indicated by red circle in (**d**)), respectively. The ZA of the SADP of the B2 is [001].

Figure 1(a) shows the TEM micrograph of the as-cast AlCoCrFeNi_2.1_ EHEA. The selected area diffraction pattern (SADP) shows the presence of ordered FCC (L1_2_) (Fig. 1(b), obtained from the green circled region in Fig. 1(a)) and ordered BCC (B2) (Fig. 1(c), obtained from the red circled region in Fig. 1(a)) phases in the lamellar morphology, as reported previously^35,36^. The average thickness of the L1_2_ (~0.57 µm) is greater than that of the B2 phase (~0.20 μm). Consequently, the volume fraction of the L1_2_ phase (~65%) is greater than the B2 phase (~35%), as determined from the EBSD phase maps^35,36^. The B2 lamellae also contain nano-sized disordered BCC precipitates^35^.

Figure 1(d) shows the microstructure of the EHEA after 90% cryo-rolling. The clear ring patterns in the SADP (Fig. 1(e) obtained from the region marked by green circle in Fig. 1(d)) reveal the formation of deformation induced ultrafine nanostructure involving various orientations. The absence of rings corresponding to the superlattice reflections confirms the disordering of the initially ordered L1_2_ phase during cryo-rolling. In contrast, the presence of clear superlattice spots in the SADP (Fig. 1(f) obtained from the region marked by the red circle in Fig. 1(d)) shows that the B2 phase maintains the ordered structure.

**Figure 2:**
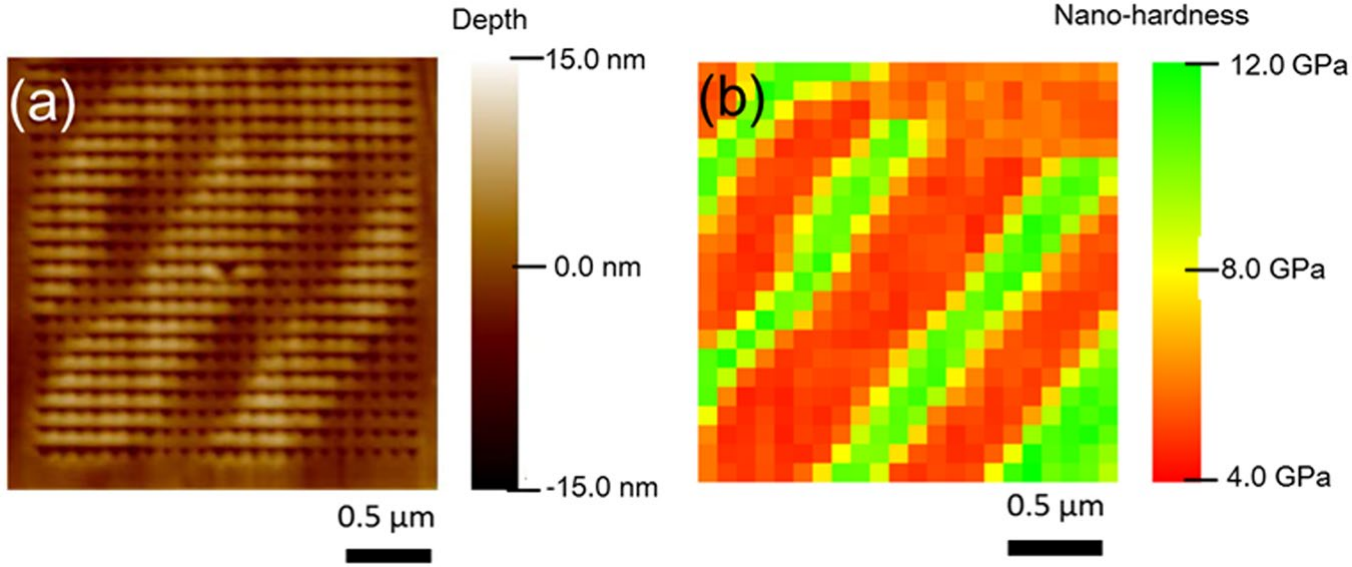
Nano-indentation (**a**) topographic and (**b**) hardness maps of the as-cast EHEA.

Figure 2(a) shows the scanning probe microscope (SPM) topographic image while Fig. 2(b) shows the corresponding hardness map of a scanned area in the as-cast material. The B2 lamellae (dark contrast regions in Fig. 2(a)) are clearly identified by their lower thickness than the FCC lamellae. The hardness map reveals much higher hardness of the B2 phase than the L1_2_ phase (Fig. 2(b)). The hardness of the B2 phase (~12 GPa) is nearly three times higher than that of the L1_2_ phase (~4 GPa) in the as-cast EHEA.

**Figure 3:**
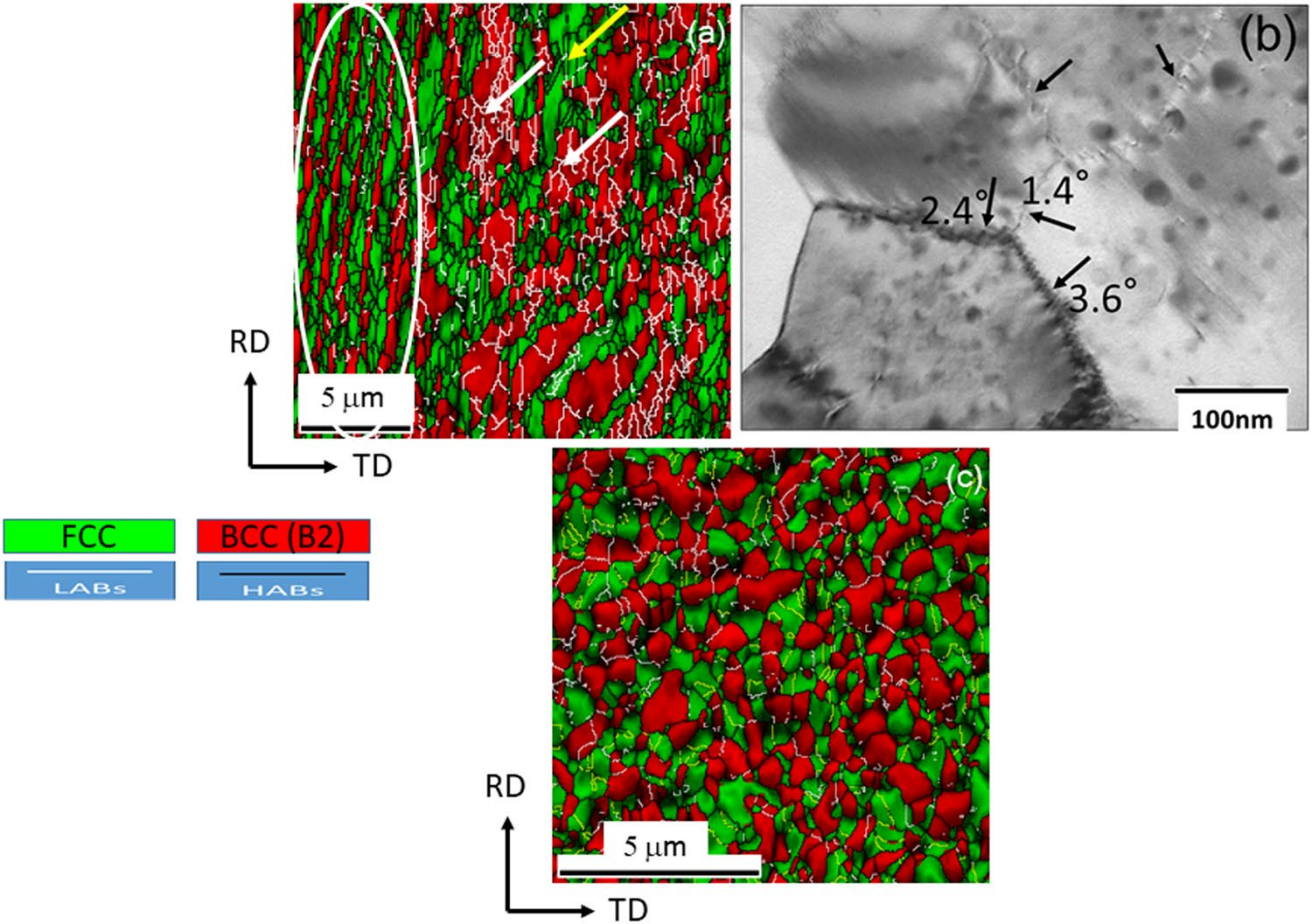
(**a**) EBSD phase map of 90% cryo-rolled EHEA after annealing at 800 °C for 1 h. The region enclosed by the ellipse in (**a**) shows a typical lamellar region while the arrows indicate coarse B2 regions. (**b**) TEM micrograph (observed along ND) shows B2 subgrains in the coarse non-lamellar region of the cryo-rolled and annealed EHEA separated by LABs (marked by arrows) along with their misorientation angles. (**c**) Shows the microduplex structure of the 90% cold-rolled EHEA after annealing at 800 °C for 1 h.

The microstructure of the 90% cryo-rolled material after annealing at 800 °C is shown in the EBSD phase map in Fig. 3(a). Interestingly, the microstructure shows remarkable heterogeneity due to the presence of the fine lamellar regions consisting of FCC (green) and B2 (red) lamellae (the region surrounded by a white ellipse), and the coarse non-lamellar regions consisting of FCC grains (regions marked by a yellow arrow) and coarse B2 phase marked by white arrows. In the lamellar region, the FCC lamellae are filled with ultrafine recrystallized grains (average size ~200–250 nm) surrounded by high angle boundaries (HABs drawn by black lines), while the B2 lamellae predominantly have only low-angle boundaries (LABs drawn by white lines). In the non-lamellar regions, ultrafine recrystallized grains of FCC with grain sizes of 200–250 nm surrounded by HABs are found, while a few coarse recrystallized FCC grains with annealing twins also exist (marked by yellow arrow). The coarse B2 in the non-lamellar regions has an average thickness of ~2.5 µm and contains profuse LAB networks, indicating unrecrystallized state of the B2 regions. This is confirmed in the TEM micrograph (Fig. 3(b)) showing B2 phase in a coarse non-lamellar region. The B2 subgrains (~200 nm) are separated by LABs (marked by arrows). The misorientation angles of selected boundaries determined using the kikuchi-line diffraction pattern analyses are shown in Fig. 3(b). The interior of the subgrains is free of dislocations, but the nano-sized precipitates present in the as-cast starting material^35^ remain undissolved.

The phase map of the cold-rolled and annealed material (Fig. 3(c)) reveals a strikingly different microstructure from that of the cryo-rolled and annealed material. Figure 3(c) shows a homogenous microduplex morphology consisting of nearly equiaxed FCC (green) and B2 (red) grains (grain sizes of ~0.5 µm). The fraction of B2 (~45%), determined from the EBSD phase map, is slightly lower than that in the cryo-rolled and annealed material (~52%). Notably, the coarse B2 grains in the cold-rolled and annealed material do not include LAB networks inside, in contrast to the B2 phase in the cryo-rolled and annealed material (Fig. 3(a)).

**Figure 4:**
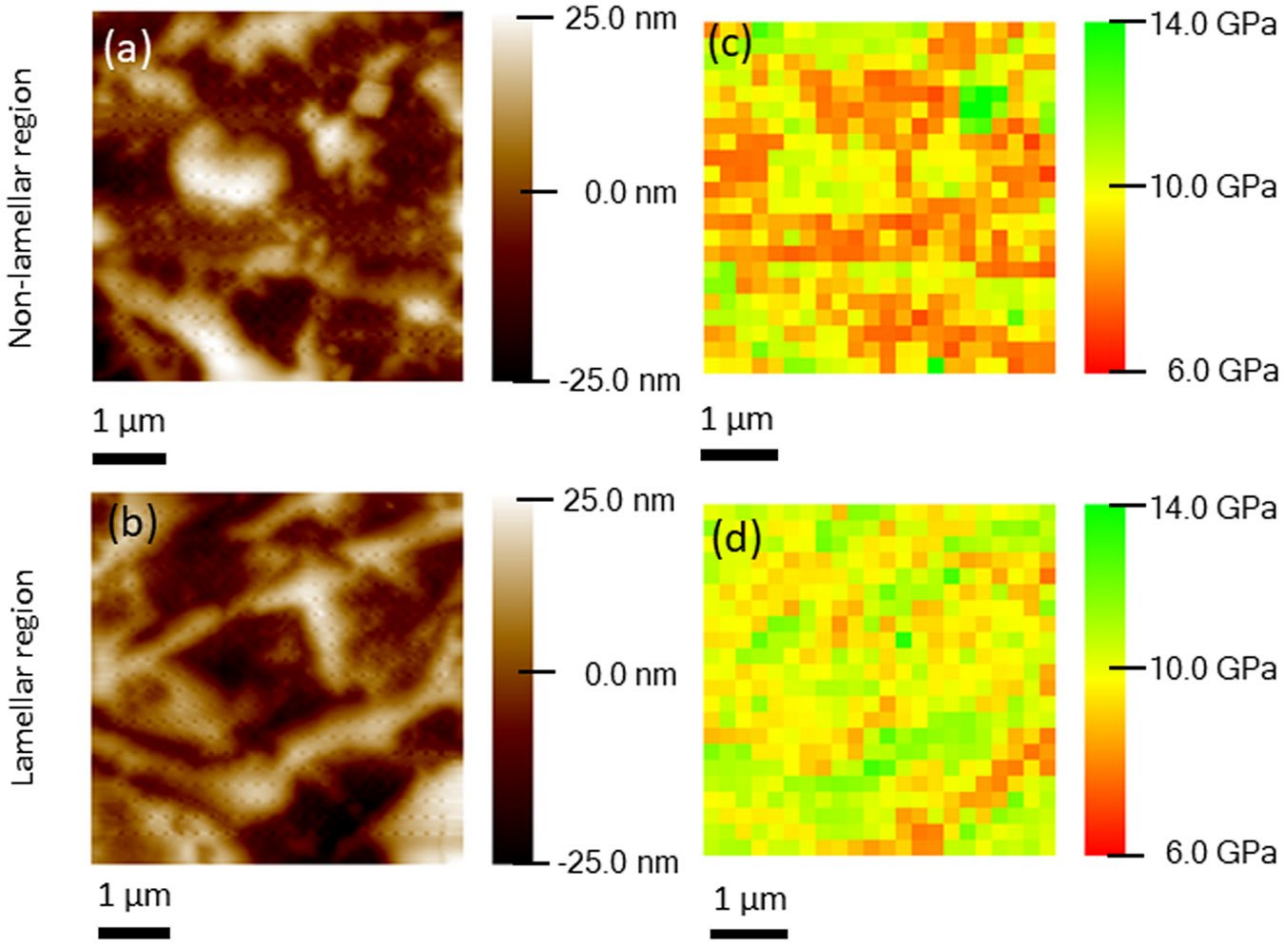
Nano-indentation topographic images of cryo-rolled and annealed specimen obtained from the (**a**) coarse non-lamellar and (**b**) lamellar regions; (**c**) and (**d**) are the corresponding hardness maps.

Figure 4(a) and (b) show typical topographic images of a coarse non-lamellar region and a fine lamellar region, respectively, in the cryo-rolled and annealed EHEA. The hardness map (Fig. 4(c)) of the coarse non-lamellar region (Fig. 4(a)) shows higher fraction of lower hardness regions (~6–8 GPa, highlighted by red) than intermediate (~10–12 GPa, highlighted in yellow) and very hard (≥12 GPa, highlighted in red) regions. The hardness map of the fine lamellar region (Fig. 4(d)) shows higher average hardness than coarse non-lamellar region (Fig. 4(c)). However, the hardness distribution in the non-lamellar region (Fig. 4(c)) covers a wider hardness spectrum. As a remarkable consequence, the cryo-rolled and annealed material has a complicated heterogeneous microstructure composed of softer regions (≤8 GPa), intermediate regions (~8–12 GPa) and much harder regions (≥12 GPa), thus covering the entire hardness spectrum. In contrast, the as-cast material consisting of soft L1_2_ and hard B2 phases with vastly different hardness (Fig. 2).

**Figure 5:**
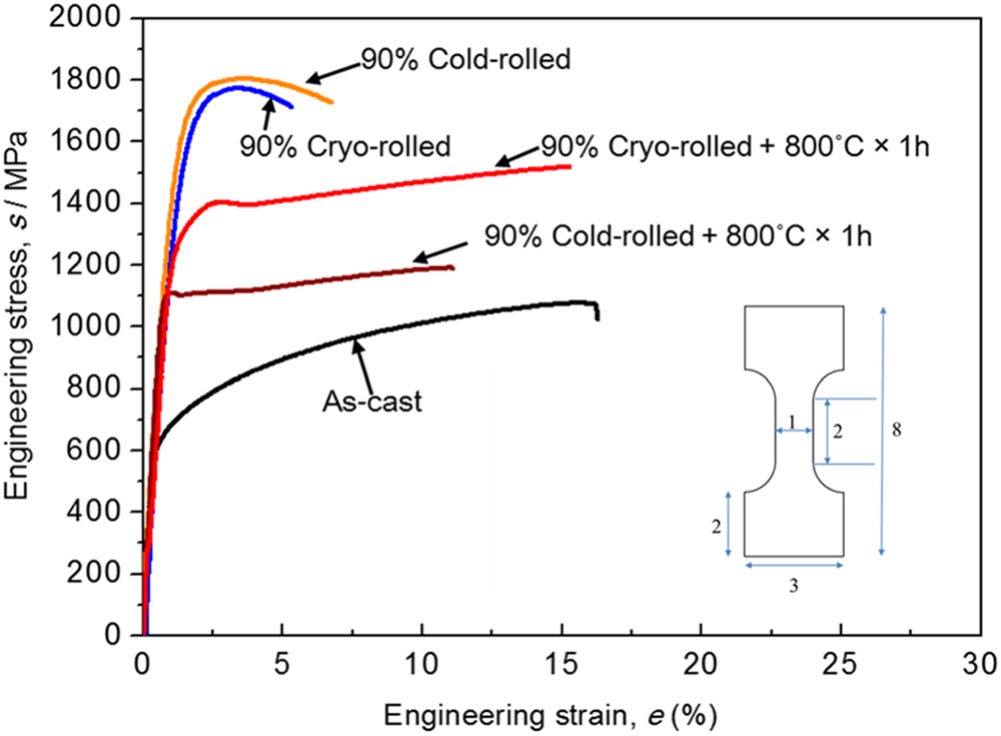
Engineering stress-strain plots of the EHEA in various heat-treated conditions. The dimensions of the tensile specimen are shown inset (all dimensions in mm).

The as-cast EHEA shows a relatively high yield strength (YS ~ 615 ± 5 MPa), ultimate tensile strength (UTS) (~1105 ± 38 MPa) and large tensile elongation (e_f_ ~ 17 ± 1%) (Fig. 5). The strength increases at the expense of ductility after cryo and cold-rolling. The EHEA specimens cold- or cryo-rolled to 90% reduction in thickness show quite similar strength and ductility. The cryo-rolled and annealed material shows a remarkable increase in YS (~1437 ± 26 MPa), UTS (~1562 ± 33 MPa) and elongation to failure (e_f_ ~ 14 ± 1%) compared with those of the cold-rolled and annealed material (YS ~ 1100 ± 8 MPa, UTS ~ 1175 ± 15 MPa and e_f_ ~ 11 ± 0.1%). Compared to the as-cast material (YS ~ 615 ± 5 MPa, UTS ~ 1105 ± 38 MPa and e_f_ ~ 17 ± 1%), the cryo-rolled and annealed material shows outstanding increase in strength but the elongation to failure remains almost unchanged.

**Figure 6:**
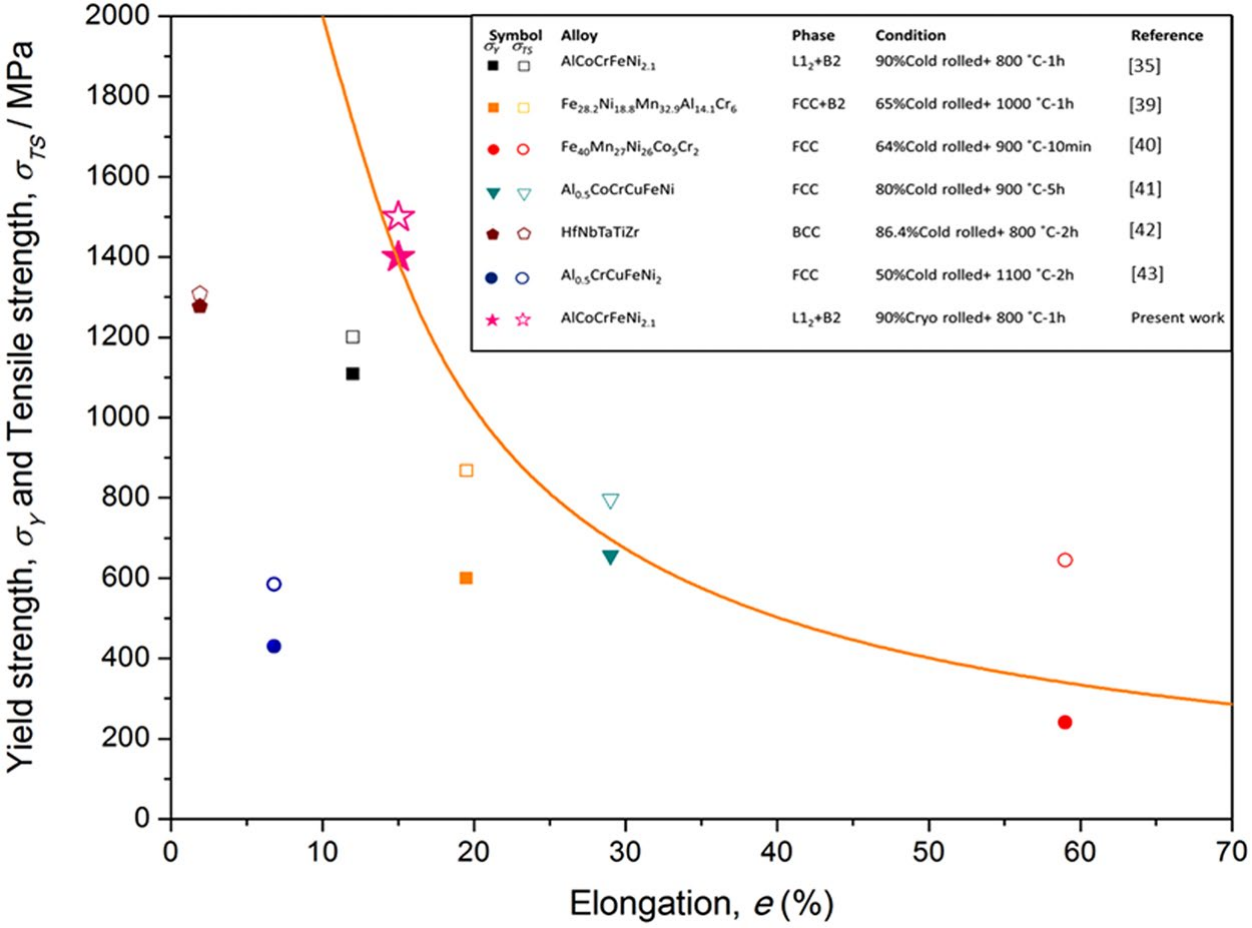
Strength-elongation plot of selected HEAs. The closed and open symbols correspond to YS and UTS, respectively. *σ*_*y*_ and *σ*_*TS*_ are YS and UTS, respectively.

The strength *vs* elongation plot (Fig. 6) compares the properties of cryo-rolled and annealed EHEA with several HEAs composed of FCC and/or BCC phases. The closed and open symbols in the plot indicate YS and UTS, respectively. The present cryo-rolled and annealed EHEA possesses the highest YS and UTS, while maintaining appreciable ductility. Figure 6 also shows the strength*elongation line corresponding to 20,000 MPa %. The present EHEA has much higher YS (and UTS) that the other two HEAs, namely Al_0.5_CoCrCuFeNi and Fe_40_Mn_27_Ni_26_Co_5_Cr_2_ which fall above this line. Evidently, the present cryo-rolled and annealed EHEA is much superior to other HEAs^39–43^ as a structural material possessing ultrahigh strength and ductility.

## Discussion

The EHEA with nano-lamellar mixture of L1_2_ and B2 phases shows disordering of the L1_2_ phase but retention of the ordered B2 structure during cryo-rolling, quite similar to the behavior observed during cold-rolling^35,36^. However, the microstructures of the cryo- and cold-rolled materials are strikingly dissimilar after annealing. The cold-rolled material shows a rather homogenous ultrafine microduplex structure after annealing due to the breakdown of lamellar deformation structure, typically observed in cold-rolled and annealed duplex alloys^44^. In sharp contrast, the cryo-rolled material after annealing shows a distinctly heterogeneous hierarchical microstructure and unprecedented tensile properties.

It is noted that hardness and flow stress of bcc materials are strongly affected by decreasing temperature (undergoing typical ductile to brittle transition) while those of FCC materials remain unaffected. These different hardness and flow stress dependencies of the two phases affect the strain-partitioning. In the starting EHEA, the B2 phase is much harder than the L1_2_ phase (Fig. 2). The relative hardness difference between the two phases (ΔH_cold_) would result in the L1_2_ phase being deformed more easily than the B2 phase at the initial stages of deformation. However, with increasing deformation at room temperatures, the B2 phase would eventually start deforming^35,39,45^. In contrast, at the cryo-rolling temperature, the hardness or the flow stress of the B2 phase is expected to increase significantly, while the hardness of the L1_2_ would be much less affected. Therefore, the relative hardness difference of the two phases during cryo-rolling (ΔH_cryo_) should be much higher than that during cold-rolling (ΔH_cryo_ ≫ ΔH_cold_). Consequently, much higher strain should be partitioned in the L1_2_ phase during cryo-rolling than that during cold-rolling. Thus, for the same deformation level, the B2 phase in the cryo-rolled EHEA accumulates much lower strain than that in the cold-rolled EHEA. The vastly different strain-partitioning patterns in cryo- and cold-rolled materials should affect the stored energy and driving force for recrystallization.

The stored energies of the two phases in the cold-rolled EHEA are sufficient for complete recrystallization resulting in the microduplex structure, similar to heavily deformed and annealed duplex steels^44^. In contrast, the significantly lower strain partitioning in the B2 phase in the cryo-rolled EHEA coupled with its ordered structure would diminish the driving force for recrystallization significantly^46^ and promote recovery. This is clearly evidenced from the presence of the distinct LAB network inside the coarse B2 sub-grains (Fig. 3(b) and (c)). In contrast, the high stored energy of the heavily deformed L1_2_/FCC phase in cryo-rolled material leads to fully recrystallized ultrafine FCC grains. This results in the formation of a unique hierarchical microstructure consisting of fine lamellar and coarse non-lamellar regions instead of a microduplex structure.

The most remarkable feature of the hierarchical microstructure of the cryo-rolled and annealed EHEA is the simultaneous increase in strength and ductility. While simultaneous enhancement in strength-ductility has been demonstrated in previously reported HEAs due to TRIP and deformation twinning^47–49^, careful TEM analysis in the present EHEA rules out the possibility of either TRIP^35,36^ or presence of extensive deformation twins^50^. Evidently, the simultaneous increase in strength and ductility of the cryo-rolled and annealed material originates from the characteristic hierarchical microstructure consisting of fine lamellar regions, ultrafine recrystallized FCC grains, coarse recrystallized FCC grains and coarse recovered B2 regions.

Recently, the role of back stress on the simultaneous enhancement in strength and ductility in heterogeneous hierarchical materials consisting of soft and hard domains has been clarified^11,51,52^. The deformation in heterogeneous materials occur in three different stages. In stage I, both soft and hard domains deform elastically similar to conventional homogeneous materials. In stage II, the soft domains will start plastically deforming while the hard domains will resist plastic deformation, leading to a mechanical incompatibility. As a result, the plastically deforming soft domains cannot deform freely. This would lead to plastic strain gradients at the domain interface, which need to be accommodated by geometrically necessary dislocations (GNDs) which should lead to significant strengthening of the soft phase. When soft domains are completely surrounded by hard domains, GNDs will pile up at the domain boundaries resulting in a high back stress. This will result in significantly higher yield strength than that predicted by the simple rule of mixtures. In stage III, both soft and hard domains will start plastically deforming but the soft domains will be subjected to a larger strain^53–55^. When the neighboring domains experience different levels of strain, strain gradients will build up near the domain boundaries. The strain gradient increases with increasing strain partitioning, producing significant back stress work hardening. In essence, the existence of strain gradients in heterogeneous materials with different hardness domains should be associated with significant back stress strengthening, which should delay necking during tensile loading and improve strength and ductility^51^.

The nano-indentation maps clearly show that the heterogeneous hierarchical microstructure in the cryo-rolled and annealed material is composed of regions consisting of soft, intermediate and hard constituents, covering a wide hardness spectrum. Thus, the microstructure of the cryo-rolled and annealed material, which gives rise to profuse domain boundaries separating domains of different hardness, appears particularly favorable to benefit from significant back stress strengthening. In contrast, the lamellar and duplex microstructures of the as-cast and cold-rolled and annealed materials, respectively are featured by a mixture of soft L1_2_/FCC and hard B2 phases, but lack the nano-scale structural and hardness hierarchy, which lead to their inferior strength-ductility combination.

It is rather interesting to note that certain intermetallic phases such as Fe_3_Al also show simultaneous increase in strength and ductility in recovered or partially recrystallized conditions (having a few recrystallized grains)^56,57^. While the mechanisms responsible for this rather interesting behavior of Fe_3_Al have not been investigated in-depth^46^, the simultaneous increase in strength and ductility of the cryo-rolled and annealed material with hierarchical microstructure composed of ultrafine recrystallized, recovered and partially recrystallized phases is evidently in excellent agreement^56,57^.

In summary, the effect of thermo-mechanical processing by cryo-rolling and annealing on microstructure and properties of nano-lamellar (L1_2_ + B2) AlCoCrFeNi_2.1_ EHEA was investigated. The cryo-rolled and annealed EHEA developed a novel hierarchical microstructure after annealing. The synergistic effect exerted by the hierarchical microstructure resulted in outstanding increase in strength without loss of ductility when compared to the as-cast as well as cold-rolled and annealed materials. The present work demonstrates the potential of cryo-rolling for developing EHEAs with outstanding strength-ductility balance.

## Materials and Methods

AlCoCrFeNi_2.1_ EHEA was prepared by arc melting in a Ti-gettered high-purity argon atmosphere using high purity elements (≥99.9%). The melt was suction-cast into a copper mold (15 mm (width) × 90 mm (length) × 3 mm (thickness)). Multi-pass cryo-rolling of the as-cast EHEA was carried out using a laboratory scale rolling mill (SPX Precision Instruments, Fenn Division, USA) up to ~90% reduction in thickness. Before and immediately after each pass, the samples were immersed in a liquid N_2_ bath for 45 minutes. The 90% cryo-rolled samples were annealed for 1 hour (h) at 800 °C and water quenched. In order to highlight the effect of cryo-rolling and annealing, comparisons were made with a 90% cold-rolled (at room temperature) specimen annealed at 800 °C for 1 h^35,36^.

Nano-indentation tests were carried out using a TI950 Tribo-indenter (Hysitron, USA) equipped with a sharp cube-corner probe (apex radius of curvature of ~40 nm). During each indentation, the load was increased linearly from 1 μN to 500 μN over a period of 0.25 seconds (sec.), kept constant for 0.25 sec., and unloaded to 1 μN over a period of 0.25 sec. before moving to the next indent position. All nano-indentation data were analyzed using the standard Oliver-Pharr method^58^. Tensile properties were determined at ambient temperature using a universal testing machine (Shimadzu, Japan) with an initial strain rate of 8.3 × 10^−4^ s^−1^.

Electron backscatter diffraction (EBSD) system (Oxford Instruments, UK) attached to a scanning electron microscope (SEM) (Carl-Zeiss, Germany; Model: SUPRA 40) and by transmission electron microscope (TEM) (JEOL 2010 operated at 200 kV) was used for microstructural studies. EBSD dataset acquired using the AztecHKL software (Oxford Instruments, UK) were analyzed by TSL-OIM^TM^ software (EDAX Inc., USA). The samples for the EBSD and TEM investigations were prepared using mechanical polishing followed by electropolishing in an electrolyte of 90% ethanol + 10% perchloric acid.
